# *Toxoplasma gondii* Clonal Strains All Inhibit STAT1 Transcriptional Activity but Polymorphic Effectors Differentially Modulate IFNγ Induced Gene Expression and STAT1 Phosphorylation

**DOI:** 10.1371/journal.pone.0051448

**Published:** 2012-12-11

**Authors:** Emily E. Rosowski, Jeroen P. J. Saeij

**Affiliations:** Massachusetts Institute of Technology, Department of Biology, Cambridge, Massachusetts, United States of America; University of Oklahoma Health Sciences Center, United States of America

## Abstract

Host defense against the parasite *Toxoplasma gondii* requires the cytokine interferon-gamma (IFNγ). However, *Toxoplasma* inhibits the host cell transcriptional response to IFNγ, which is thought to allow the parasite to establish a chronic infection. It is not known whether all strains of *Toxoplasma* block IFNγ-responsive transcription equally and whether this inhibition occurs solely through the modulation of STAT1 activity or whether other transcription factors are involved. We find that strains from three North American/European clonal lineages of *Toxoplasma*, types I, II, and III, can differentially modulate specific aspects of IFNγ signaling through the polymorphic effector proteins ROP16 and GRA15. STAT1 tyrosine phosphorylation is activated in the absence of IFNγ by the *Toxoplasma* kinase ROP16, but this ROP16-activated STAT1 is not transcriptionally active. Many genes induced by STAT1 can also be controlled by other transcription factors and therefore using these genes as specific readouts to determine *Toxoplasma* inhibition of STAT1 activity might be inappropriate. Indeed, GRA15 and ROP16 modulate the expression of subsets of IFNγ responsive genes through activation of the NF-κB/IRF1 and STAT3/6 transcription factors, respectively. However, using a stable STAT1-specific reporter cell line we show that strains from the type I, II, and III clonal lineages equally inhibit STAT1 transcriptional activity. Furthermore, all three of the clonal lineages significantly inhibit global IFNγ induced gene expression.

## Introduction

The cytokine interferon-gamma (IFNγ) and the transcription factor it activates, signal transducer and activator of transcription (STAT) 1, are critical to host defense against the obligate intracellular parasitic pathogen *Toxoplasma gondii*; mice deficient in elements of this pathway are acutely susceptible to *Toxoplasma* infection [1]–[3]. Activated STAT1 induces the expression of genes with gamma activated sequence (GAS) elements in their promoters, including the interferon regulatory factor (IRF) 1 transcription factor. STAT1 and IRF1 together induce a broad transcriptional program including effector mechanisms that mediate pathogen destruction or inhibition of pathogen growth [4].

However, *Toxoplasma* infection can inhibit IFNγ induced gene expression in host cells, and was first shown to inhibit the basal and IFNγ induced expression of MHC class II molecules, in a variety of cell types [5]–[7]. Since then, *Toxoplasma* has also been shown to inhibit the expression of IRF1 [8], [9], class II transactivator (CIITA) [7]–[9], inducible nitric oxide synthase (iNOS/NOS2) [10], [11], interferon inducible GTPase 1 (IIGP1) [12], and chemokine (C-X-C motif) ligand 9 (MIG/CXCL9) [12]. This inhibition occurs in a variety of cell types, including human foreskin fibroblasts (HFF), human glioblastoma cells, murine bone marrow-derived macrophages (BMDM), RAW264.7 murine macrophages, murine dendritic cells, and murine microglial cells. Microarray analyses showed that *Toxoplasma* infection can dysregulate the entire IFNγ induced gene expression program in both HFFs [13] and BMDMs [14].

*Toxoplasma* infects virtually all warm-blooded animals, including ∼30% of the worldwide human population [15]. Many different strains of *Toxoplasma* have been isolated from various hosts, and in North America and Europe the majority of *Toxoplasma* isolates from humans and livestock belong to three main clonal lineages: types I, II, and III [16]. These strains differ in the modulation of multiple host cell signaling pathways through polymorphic effectors secreted into the host cell from rhoptry and dense granule organelles [17]. While all of these strains can inhibit the expression of at least certain IFNγ induced genes, it is unknown whether all of the strains can inhibit global IFNγ induced gene expression and STAT1 transcriptional activity, or whether the degree of inhibition varies between *Toxoplasma* strains.

Many STAT1 regulated genes can be induced or repressed by other transcription factors, for example NF-κB and STAT3/6, and such genes might not be the best readouts to determine if *Toxoplasma* specifically inhibits STAT1 activity. Another question that is still unanswered is whether the activation of other transcription factors by *Toxoplasma* affects the IFNγ response. Specifically, the modulation of STAT3/6 and NF-κB transcription factors through the effector proteins ROP16 [18] and GRA15 [19], respectively, might affect this response.

The polymorphic rhoptry kinase ROP16 from type I and III strains activates the transcription factors STAT3 and STAT6 [18], [20], [21]. In STAT3 deficient cells [22] or cells with STAT6 knocked down [23], increased transcription of STAT1 target genes has been found, suggesting that STAT3 and STAT6 can antagonize STAT1 activity. STAT6 can also compete for promoter sites with STAT1 [24]. It is therefore possible that the activation of STAT3/6 by ROP16 helps to suppress IFNγ induced signaling.

SOCS family proteins are important negative regulators of the IFNγ response and in *Socs1* ^−/−^ BMDM, *Toxoplasma* could not inhibit the IFNγ response as well as in wild-type BMDM [12]. ROP16 is a strong activator of SOCS family gene expression; in murine BMDM, *Socs1*, *2*, and *3* are more than 10-fold induced by ROP16 expression [25]. It is therefore possible that ROP16 plays a role in the inhibition of the IFNγ response through the induction of *Socs* genes. Furthermore, the expression of genes that are co-regulated by both STAT1 and STAT3/6 transcription factors could also be affected by ROP16. If the expression level of such a gene was chosen to measure STAT1 activity, incorrect conclusions might be drawn.

The type II version of the dense granule protein GRA15 activates the host cell NF-κB pathway [19]. NF-κB also co-regulates many of the same genes as STAT1, and NF-κB activation combined with STAT1 activation synergistically induces IRF1 expression and activity [26]. It is therefore possible that strains possessing an active copy of GRA15 do not inhibit IFNγ induced gene expression as well as other strains, or differentially inhibit subsets of IFNγ responsive genes. In fact, a type II Δ*gra15* strain grows faster *in vivo* than a type II strain [19], and *GRA15* corresponds to a *Toxoplasma* virulence locus [19], [27].

In this report we show that the polymorphic effectors GRA15 and ROP16 do contribute to strain differences in the modulation of IFNγ-STAT1 signaling. Type II GRA15 induces the expression of IRF1, which can induce a subset of IFNγ responsive genes. ROP16 induces the tyrosine phosphorylation and nuclear translocation of STAT1 but this STAT1 is not transcriptionally active. In spite of these differences, type I, II, and III parasites can all inhibit global IFNγ induced transcription as determined by microarray analysis. Because many STAT1-regulated genes can be controlled by other transcription factors we directly measured STAT1 activity with a stable STAT1-specific reporter cell line and find that neither GRA15 nor ROP16 affects the ability of *Toxoplasma* to inhibit STAT1 transcriptional activity.

## Materials and Methods

### Parasites and Cells

Parasites were maintained *in vitro* by serial passage on monolayers of human foreskin fibroblasts (HFFs), as described previously [19]. RH or GT1 were used as representative type I strains, Pru or ME49 as representative type II strains and CEP or VEG as representative type III strains. A Pru strain engineered to express firefly luciferase and GFP (Pru Δ*hxgprt* A7) [28], and CEP and RH strains engineered to express clickbeetle luciferase and GFP (CEP *hxgprt* ^−^ C22 and RH 1–1) [29] have been described previously. An RH Δ*rop16* strain (provided by John Boothroyd, Stanford University) [21], an RH Δ*rop16* strain expressing firely luciferase and GFP (clone 1A2) [25], a PruA7 *ROP16_I_* strain [25], and Pru Δ*gra15*, PruA7 Δ*gra15*, and RH *GRA15_II_* strains [19] have been described previously. HFFs (provided by John Boothroyd, Stanford University) and RAW264.7 (ATCC) cells were grown as described previously [19], [25]. 293FT and HEK293 cells were grown with additional 10 mM HEPES. U3A STAT1-null cells [30], [31] (provided by George Stark, Cleveland Clinic Foundation Research Institute, Ohio) were grown with 10 mM HEPES, 1 mM sodium pyruvate, and MEM non-essential amino acids. All parasite strains and cell lines were routinely checked for *Mycoplasma* contamination and it was never detected.

### Reagents

Antibodies against IRF1 (BD Biosciences #612046), phospho-STAT1^Tyr701^ 58D6 (Cell Signaling #9167), phospho-STAT1^Ser727^ (Cell Signaling #9177), total STAT1α p91 (C-24) (Santa Cruz #345), GAPDH (6C5) (Santa Cruz #32233), and *Toxoplasma* surface antigen (SAG)-1 (kindly provided by John Boothroyd, Stanford University) were used in immunofluorescence and Western blot assays. Secondary antibodies coupled with either Alexa Fluor 488 or Alexa Fluor 594 (Molecular Probes) for immunofluorescence assay or conjugated to peroxidase (Kirkegaard & Perry Laboratories) for Western blots were used. Recombinant human IFNγ (100 U/ml, AbD serotec) and murine IFNγ (100 U/ml, Calbiochem) were used to stimulate cells.

### Immunofluorescence Assay

Immunofluorescence assay was performed as described previously [19]. Briefly, cells were fixed with 3% formaldehyde, permeabilized with 100% ethanol and/or 0.2% Triton-X 100, and blocked with 3% BSA and 5% goat serum. Coverslips were incubated with primary antibody at 4°C, and fluorescent secondary antibodies and Hoechst dye were used for antigen and DNA visualization, respectively. Photographs were taken using NIS-Elements software (Nikon) and a digital camera (Coolsnap EZ; Roper Scientific) connected to an inverted fluorescence microscope (model eclipse Ti-S; Nikon). Quantification of nuclear signal was performed by randomly selecting cells in each condition and measuring the average signal intensity per nucleus using the NIS-Elements software and Hoechst dye to define nuclei. The minimum number of cells measured is indicated in the figure legends for each experiment.

### Western Blot

Western blots were performed as described previously [19]. Briefly, HFFs were left uninfected or infected with RH Δ*hxgprt*, RH 1–1, RH Δ*rop16*, RH Δ*rop16* 1A2, Pru Δ*hxgprt* A7, or CEP *hxgprt^ -^* C22 parasites for three hours. Samples were subsequently stimulated with human IFNγ for one hour, or left unstimulated, and then lysed in buffer containing sodium dodecyl sulfate (SDS) and either β-mercaptoethanol (βME) or dithiothreitol (DTT). After immunoblotting, membranes were stripped with boiling 2% SDS and 0.7% βME and reprobed.

### Reporter Cell Line Construction

A GAS (TR027PA-1, 5′-AGTTTCATATTACTCTAAATC -3′) pGreenFire1 (pGF1) lentiviral reporter vector containing a Neo selection cassette and a minimal CMV promoter followed by four tandem consensus GAS sites driving the expression of Firefly luciferase was purchased from System Biosciences. The vector was co-transfected into 293FT cells with vectors containing gag, pol, and VSV-G proteins using FuGENE reagent (Roche) according to the manufacturer’s protocol. Supernatant containing virus was collected two and three days after transfection, filtered with a 0.45 µm surfactant-free cellulose acetate filter (Nalgene), and added to HEK293 cells (ATCC) with 8 µg/ml polybrene (Sigma). HEK293 cells containing the pGF1 construct were then selected with 750 µg/ml Geneticin (Invitrogen). Cells were cloned by limiting dilution and were confirmed to be responsive to IFNγ but not to IFNβ, TNFα, or IL4.

### Luciferase Assay

HEK293 pGF1-GAS cells were plated in 96-well plates, 3.5–4×10^4^ cells/well, and grown for 4–20 hours. Cells were then infected with RH Δ*hxgprt*, RH Δ*rop16*, GT1, Pru Δ*hxgprt*, Pru Δ*gra15*, ME49, CEP Δ*hxgprt*, or VEG parasites at varying MOIs for 1–4 hours, and subsequently stimulated with human IFNγ for 12–24 hours. Cells were lysed with 20 µl Cell Culture Lysis Reagent (Promega) containing 1× protease inhibitors (Roche), and plates were frozen at −80°C. Luciferase activity in plates was detected using a Varioskan Flash Reader (Roche) after addition of 100 µl Luciferase assay substrate (Promega), according to the manufacturer’s instructions. Data were normalized to the uninfected, unstimulated sample and averaged over at least two experiments per condition.

### Microarray

1.5×10^6^ RAW264.7 cells were plated in 6-well plates and grown for 24 hours. The cells were then left uninfected or infected with RH 1–1, Pru Δ*hxgprt* A7, or CEP *hxgprt* ^-^ C22 parasites at an MOI ∼5 for 18 hours and subsequently stimulated with murine IFNγ for six hours. The RH infection was done at one time and Pru and CEP infections were done together at a later time. Uninfected controls were included for both sets of infections. RNA was isolated and microarray analysis, including analysis with the DiRE server, was performed as described previously [19], with Mouse 430A 2.0 Affymetrix gene chips. Microarray data has been uploaded to NCBI Gene Expression Omnibus and are accessible through GEO Series accession number GSE34913.

### Plaque Assay

For Western blot, luciferase reporter, and microarray assays, a plaque assay was done to determine the viability of each strain and the actual MOI. One hundred parasites per well were added to confluent HFFs in a 24-well plate and were incubated undisturbed for 5–7 days at 37°C, and the number of plaques was counted. Samples with similar MOIs were then picked for analysis.

## Results

### A Type II Strain Activates IRF1 via GRA15 and NF-κB

**Figure 1:**
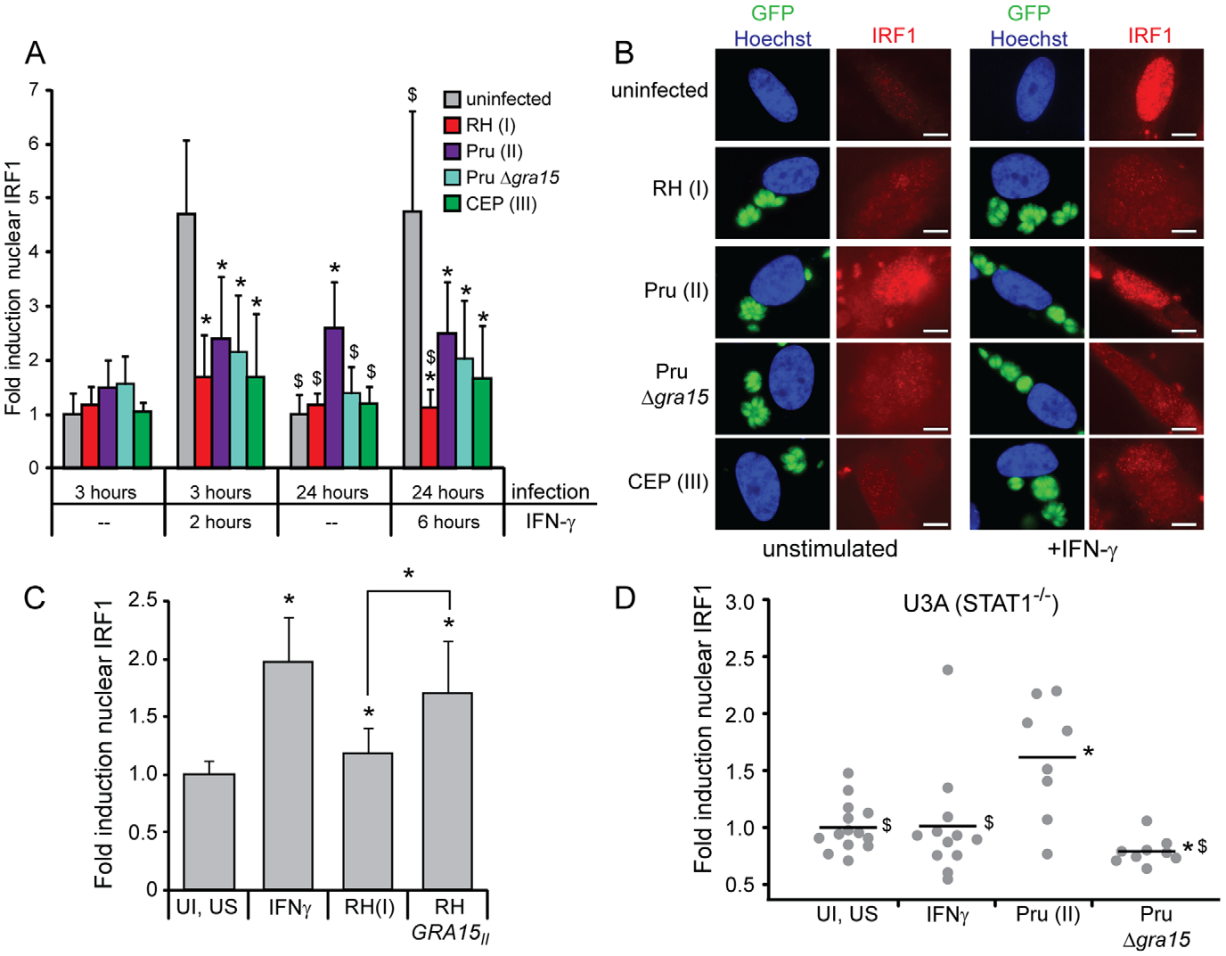
Type II GRA15 affects the expression of IRF1. HFFs were infected with RH(I), RH *GRA15_II_*, Pru(II), PruΔ*gra15*, or CEP(III) parasites and/or stimulated with 100 U/ml IFNγ, subsequently fixed and permeabilized and stained with an antibody against IRF1 (red) and with Hoechst dye (blue, nucleus). **A, B.** HFFs were infected for three or 24 hours with GFP-expressing parasites (green), or left uninfected, and stimulated with IFNγ for the last two or six hours, or left unstimulated. Nuclear localization of IRF1 was quantified in at least 12 randomly selected cells per condition and normalized to uninfected, unstimulated cells (A) and a representative cell for each condition is shown (B). Scale bar represents 10 µm. This experiment was performed three times with similar results. Data and standard deviation from one representative experiment are shown. Asterisk (*) indicates p<0.05 compared to uninfected cells, dollar sign ($) indicates p<0.05 compared to type II infected cells. **C.** HFFs were infected with an RH(I) or RH *GRA15_II_* strain, left uninfected, or left uninfected and stimulated with IFNγ for 24 hours. Nuclear localization of IRF1 was quantified in at least 30 randomly selected cells and normalized to uninfected, unstimulated cells. Data and standard deviation from one experiment are shown. Asterisk (*) indicates p<0.05 compared to uninfected cells or as represented by brackets. **D.** U3A STAT1-deficient cells were infected with Pru(II) or PruΔ*gra15* parasites for 20 hours, left uninfected, or stimulated with IFNγ for 1 hour. Nuclear localization of IRF1 was quantified in at least 8 randomly selected cells per condition, and normalized to uninfected, unstimulated cells. This experiment was performed twice with similar results, data from one representative experiment are shown. Asterisk (*) indicates p<0.05 compared to uninfected cells, dollar sign ($) indicates p<0.05 compared to type II infected cells.

Infection of HFFs with a type II Pru strain of *Toxoplasma* was previously shown to induce the expression of 46 genes that were also defined as IFNγ regulated [13], raising the possibility that type II strains are not as good at inhibiting IFNγ induced gene expression as other clonal lineages. To compare the ability of type I, II, and III strains to inhibit the IFNγ response we pre-infected HFFs with RH(I), Pru(II), or CEP(III) strains, or left cells uninfected, and subsequently stimulated the cells with IFNγ or left them unstimulated. We then visualized and quantified the amount of IRF1 in the nucleus by immunofluorescence. IRF1 is a primary response gene induced directly by STAT1 upon IFNγ stimulation. After three hours of infection, with IFNγ stimulation for the last two hours, cells pre-infected with either RH(I), Pru(II), or CEP(III) all had significantly lower levels of IRF1 in their nuclei than uninfected cells (Fig. 1A, B), as was previously seen for type I, II, and III strains [8], [9], [13]. However, after 24 hours of infection, with IFNγ stimulation for the last six hours, while RH(I), Pru(II), and CEP(III) infection all significantly inhibited IRF1 expression compared to uninfected cells, cells pre-infected with a Pru(II) strain had significantly higher IRF1 in their nuclei than cells pre-infected with a RH(I) strain (Fig. 1A, B). Cells pre-infected with a Pru(II) strain also had higher levels of IRF1 than cells pre-infected with a CEP(III) strain but this difference was not statistically significant. These data suggest that a Pru(II) strain does not inhibit IRF1 expression as well as RH(I) or CEP(III) strains.

We next determined IRF1 levels after infection in the absence of IFNγ. In unstimulated cells infected with Pru(II) for 24 hours, we find ∼2.5 fold higher nuclear IRF1 levels than in uninfected cells or cells infected with either RH(I) or CEP(III) (Fig. 1A, B). These data suggest that the different IRF1 protein levels observed in Pru(II) and RH(I) infected cells after IFNγ treatment may not be due to differences in the ability of these strains to inhibit IFNγ induced gene expression but instead due to the induction of IRF1 by Pru(II) infection, independently of IFNγ.

Although IRF1 is primarily induced by interferons it also has three NF-κB binding sites in its promoter [4] and these are important for the synergistic induction of genes by IFNγ and TNFα [26]. Type II parasites activate NF-κB-mediated transcription via the dense granule protein GRA15 [19] and we hypothesized that GRA15-mediated NF-κB activation could drive the expression of IRF1. To test this hypothesis, we also infected HFFs with a PruΔ*gra15* strain (Fig. 1A, B). After 24 hours of infection, this strain induced significantly less IRF1 protein than a Pru strain (p<0.001) and PruΔ*gra15* infected cells have similar IRF1 levels as cells infected with RH(I) and CEP(III) strains which possess inactive copies of GRA15 [19]. An RH(I) strain ectopically expressing a type II copy of GRA15 also induced IRF1 expression in HFF host cells (Fig. 1C).

To determine whether this GRA15-mediated activation of IRF1 is dependent on STAT1, we infected U3A STAT1-deficient cells [30], [31] with either Pru(II) or PruΔ*gra15* parasites, or stimulated the cells with IFNγ. While IFNγ treatment, which relies on STAT1 signaling, does not activate IRF1 expression in these cells, infection with Pru(II) parasites does, and this activation is again dependent on the presence of GRA15 (Fig. 1D), demonstrating that the GRA15-mediated induction of IRF1 is through a different transcription factor. GRA15 is known to activate the NF-κB p65 transcription factor [19], and since it is also known that NF-κB can activate the expression of IRF1 [4], [26], we hypothesized that GRA15 was inducing IRF1 through the activation of NF-κB p65. Indeed, in a previous microarray analysis [19], while *Irf1* transcript was induced by infection of wild-type MEFs with GRA15-expressing Pru(II) parasites, infection with this strain did not induce *Irf1* transcript in p65^−/−^ MEFs, strongly suggesting that induction of IRF1 expression by GRA15 is through the NF-κB p65 transcription factor.

IRF1 is itself a transcription factor and to test whether GRA15 might be responsible for more than just the expression of IRF1, but also the expression of other IFNγ regulated genes that were found to be induced by Pru infection [13], we re-analyzed the microarray data from which this observation was made. We found 775 oligonucleotide probes that were at least two-fold induced in HFFs by IFNγ treatment and by Pru infection. These 775 probes correspond to 374 genes also present in a microarray analysis of HFFs infected with GRA15-deficient and GRA15-overexpressing *Toxoplasma* strains [19]. Of these 374 genes, 43 were previously found to be at least two-fold GRA15-regulated in at least one parasite genetic background [19], a significant enrichment (p = 0.03, hypergeometric test), indicating that GRA15 does induce the expression of a subset of IFNγ responsive genes (Data S1). Therefore, while type I, II, and III *Toxoplasma* strains can all inhibit the IFNγ induced expression of IRF1, type II strains also induce IRF1 expression, independently of STAT1, most likely through GRA15-mediated activation of NF-κB p65. This IRF1 induction also leads to the expression of a small subset of other IFNγ responsive genes.

### *Toxoplasma* Infection Affects STAT1 Phosphorylation

After IFNγ treatment, STAT1 is tyrosine phosphorylated in the cytoplasm which allows it to traffic to the nucleus. Most recently, it was shown that infection of cells with type II Pru [13] or NTE parasites [9], [14] does not inhibit IFNγ-induced STAT1 trafficking into the nucleus. Previously however, the nuclear translocation of STAT1 was reported to be inhibited by type II (NTE) *Toxoplasma* infection [8]; the tyrosine phosphorylation of STAT1 was reported to be inhibited by type I (BK) infection [12], and type I (RH), type II (Pru), and type III (CL14) *Toxoplasma* strains were suggested to cause dephosphorylation of STAT1 in the nucleus [13].

**Figure 2:**
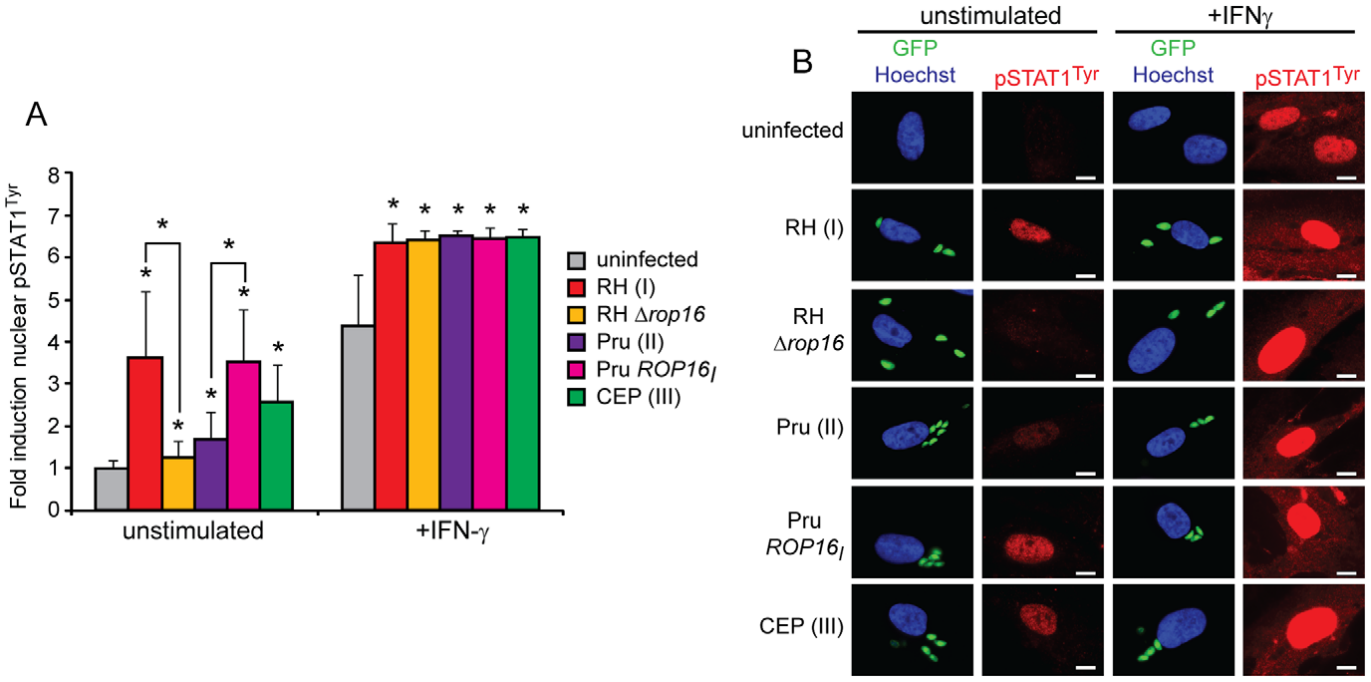
ROP16 activates STAT1 tyrosine phosphorylation and nuclear translocation. **A, B.** HFFs were infected with a GFP (green) expressing RH(I), RHΔ*rop16*, Pru(II), Pru *ROP16_I_*, or CEP(III) strain, or left uninfected, for 3 hours, and 100 U/ml IFNγ was added for the last two hours of infection, or cells were left unstimulated. Cells were fixed, permeabilized, and stained with anti-phospho-STAT1^Tyr^ (red) and Hoechst dye (nucleus, blue). Nuclear localization of phospho-STAT1^Tyr^ was quantified in at least 30 randomly selected cells infected with at least three parasites (A) and a representative cell for each condition is shown (B). Scale bar represents 10 µm. This experiment was performed for each condition at least two times with similar results. Data and standard deviation from one representative experiment are shown. Asterisk (*) indicates p<0.05 compared to uninfected cells or as indicated by brackets.

To determine if there are strain differences in the effect of infection on IFNγ-induced STAT1 phosphorylation and localization, we infected HFFs for one hour with either RH(I), Pru(II), or CEP(III) parasites, subsequently stimulated the cells for two hours with IFNγ, and quantified STAT1 tyrosine phosphorylation and nuclear translocation by immunofluorescence (Fig. 2A, B). Quantification of the immunofluorescence signal revealed that levels of IFNγ induced nuclear phospho-STAT1^Tyr^ were actually higher in infected cells compared to uninfected cells (Fig. 2A). We therefore find that none of the tested *Toxoplasma* strains inhibit the tyrosine phosphorylation or nuclear accumulation of phospho-STAT1^Tyr^ after IFNγ treatment, which agrees with the majority of previous results.

Since we observed higher levels of phospho-STAT1^Tyr^ in infected cells as compared to uninfected cells after IFNγ stimulation, we wondered whether infection with type I, II, or III parasites induces nuclear phospho-STAT1^Tyr^ in the absence of IFNγ. We infected HFFs for three hours with either RH(I), Pru(II), or CEP(III) parasites, and quantified STAT1 tyrosine phosphorylation and nuclear translocation by immunofluorescence. Indeed, we observed nuclear phospho-STAT1^Tyr^ in unstimulated cells infected with three or more RH(I) or CEP(III) parasites, and to a lower level in cells infected with three or more Pru(II) parasites (Fig. 2B). We quantified this signal in cells infected with three or more parasites and find that infection results in a significant increase in nuclear phospho-STAT1^Tyr^ levels in RH(I) and CEP(III) infected cells (Fig. 2A). Pru(II) infection also significantly induces phospho-STAT1^Tyr^, although not as highly as RH(I) or CEP(III) parasites (Fig. 2A).

We next sought to determine what parasite factor induces phospho-STAT1^Tyr^ after host cell infection. It is known that the secreted rhoptry kinase ROP16 from type I and III strains can directly tyrosine phosphorylate STAT3 and STAT6 [20], [21]. The first 700 amino acid residues of STATs 1–6 share up to 40% identity [32], raising the possibility that ROP16 also induces the tyrosine phosphorylation of STAT1. To determine if ROP16 is required for the tyrosine phosphorylation of STAT1 by RH(I) parasites in non-IFNγ-stimulated conditions, we also infected HFFs with RHΔ*rop16* parasites and again visualized phospho-STAT1^Tyr^ nuclear accumulation by immunofluorescence. As compared to cells infected with RH(I) parasites, cells infected with RHΔ*rop16* parasites had significantly less phospho-STAT1^Tyr^ in their nuclei, with levels almost as low as in uninfected cells (Fig. 2A, B). We next infected HFFs with a Pru strain that overexpresses the type I copy of *ROP16*. The ectopic expression of ROP16_I_ in a type II background led to an increase in phospho-STAT1^Tyr^ after infection (Fig. 2A, B). However, deletion of *ROP16* from a type I parasite background or overexpression of *ROP16_I_* in a type II parasite background did not affect the level of phospho-STAT1^Tyr^ in infected cells after IFNγ treatment, indicating that the increase in phospho-STAT1^Tyr^ in infected cells after IFNγ stimulation occurs independently of ROP16 (Fig. 2A, B). Together, these results demonstrate that in the absence of IFNγ, ROP16 can induce the tyrosine phosphorylation of STAT1.

### ROP16 Activated STAT1 is not Transcriptionally Active

**Figure 3:**
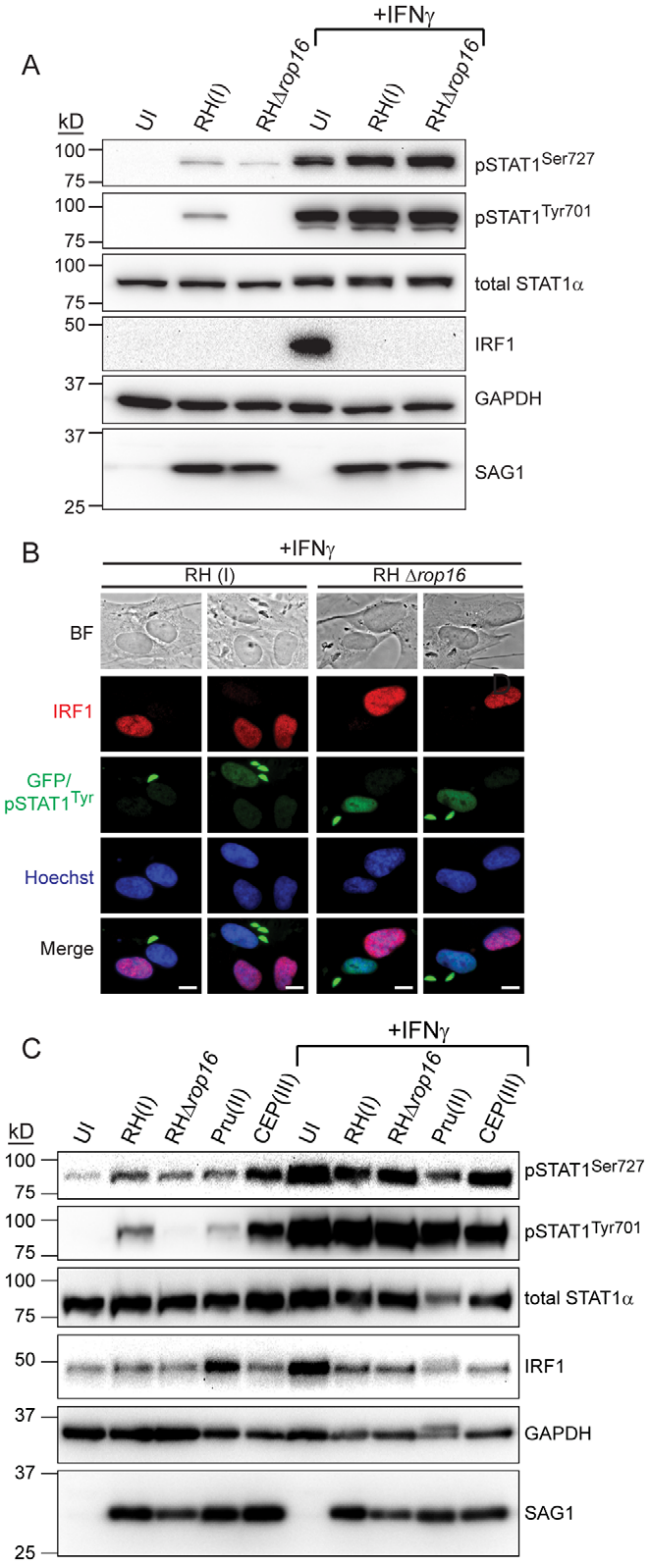
ROP16-activated STAT1 is not transcriptionally active. A. HFFs were infected with RH(I) or RHΔ*rop16* parasites at an MOI ∼7, or left uninfected, for four hours. Cells were stimulated, or not, with 100 U/ml human IFNγ for the last hour of infection and cell lysates were collected, run on an SDS-PAGE gel, and Western blotted for phospho-STAT1^Ser727^, phospho-STAT1^Tyr701^, total STAT1α, IRF1, GAPDH (host cell loading control) and SAG1 (parasite loading control). This experiment has been performed three times with similar results. **B.** HFFs were infected with RH(I) or RHΔ*rop16* parasites for four hours, stimulated with 100 U/ml IFNγ for the last two hours of infection, fixed, and stained with anti-IRF1 (red), anti-phospho-STAT1^Tyr^ (green), and Hoechst dye (nucleus, blue). Parasites also express GFP (green). Scale bar represents 10 µm. This experiment was performed three times with similar results. **C.** HFFs were infected with RH(I), RHΔ*rop16*, Pru(II), or CEP(III) parasites at an MOI ∼1, or left uninfected, for four hours. Cells were stimulated, or not, with 100 U/ml human IFNγ for the last hour of infection and cell lysates were collected, run on an SDS-PAGE gel, and Western blotted for phospho-STAT1^Ser727^, phospho-STAT1^Tyr701^, total STAT1α, IRF1, GAPDH (host cell loading control) and SAG1 (parasite loading control). This experiment has been performed two times with similar results.

Our results indicate that ROP16 can directly activate STAT1 and it is therefore possible that strains with an active ROP16 (I and III) might be less efficient in inhibiting IFNγ mediated STAT1 activation. On the other hand, ROP16 also activates STAT3 and STAT6, both of which can induce *SOCS* gene expression, which might inhibit IFNγ-STAT1 signaling [33], [34]. Indeed, we previously showed that *Socs1*, a potent inhibitor of the IFNγ-STAT1 signaling pathway, is one of the host genes most highly induced by ROP16 expression [25]. To determine if ROP16 might play a role in the modulation of the IFNγ response, we infected HFFs with an RH(I) or RHΔ*rop16* strain for three hours, or left cells uninfected, and subsequently stimulated with IFNγ for one hour, or left cells unstimulated, and analyzed IRF1 protein levels by Western blot. While only RH(I) infection induced the tyrosine phosphorylation of STAT1 in the absence of IFNγ (Fig. 3A), RH(I) and RHΔ*rop16* parasites both completely inhibited IFNγ induced IRF1 expression (Fig. 3A), indicating that ROP16 induced phospho-STAT1^Tyr^ is not transcriptionally active and that ROP16 is not required for the ability of *Toxoplasma* infection to block IFNγ induced STAT1 mediated gene expression. These results were confirmed in an immunofluorescence assay. After two hours of infection with either RH or RHΔ*rop16* parasites, HFFs were subsequently stimulated with IFNγ for two hours, cells were fixed and permeabilized, and IRF1 expression and STAT1 tyrosine phosphorylation were visualized. As seen previously (Fig. 2A, B), after IFNγ treatment, cells infected with either RH or RHΔ*rop16* had higher nuclear phospho-STAT1^Tyr^ than uninfected cells (Fig. 3B). But, as in the Western blot results (Fig. 3A), both strains clearly inhibited IFNγ induced IRF1 expression (Fig. 3B).

In addition to STAT1 tyrosine phosphorylation at residue 701, which is required for dimerization and nuclear translocation, STAT1 also must be serine phosphorylated at residue 727 for full transcriptional activity [35]. We wondered whether ROP16 or type I, II, or III strains of *Toxoplasma* affect this serine phosphorylation. It was previously shown that infection with a Pru(II) strain of *Toxoplasma* does not interfere with IFNγ induced serine phosphorylation of STAT1 in HFFs [13], but this has not been shown for any type I or III strains. We infected HFFs with an RH(I), RHΔ*rop16*, Pru(II), or CEP(III) strain, or left cells uninfected, for three hours, subsequently stimulated cells with IFNγ for one hour, or left cells unstimulated, and analyzed lysates by Western blot. We first blotted for IRF1 as a control to confirm that infection with any of these strains inhibited the IFNγ induced accumulation of IRF1 (Fig. 3C), as we have shown by immunofluorescence (Fig. 1A, B, Fig. 3B). IRF1 was not inhibited as strongly in this infection as compared to the previous Western blot (Fig. 3A) due to a lower MOI. Additionally, Pru(II) infection alone led to the induction of IRF1 protein, in concordance with previous immunofluorescence experiments (Fig. 1A,B). We next analyzed STAT1 phosphorylation in these lysates. Consistent with our immunofluorescence data (Fig. 2A,B), infection with RH(I) or CEP(III) led to a high level of phospho-STAT1^Tyr^ as compared to uninfected cells while a Pru(II) strain also induced phospho-STAT1^Tyr^ but to a lesser extent (Fig. 3C). Deletion of *ROP16* from RH almost completely abolished this tyrosine phosphorylation (Fig. 3C). In addition, none of these strains inhibited the IFNγ induced accumulation of phospho-STAT1^Tyr^. Looking next at STAT1 serine phosphorylation, we found that infection with any of the *Toxoplasma* strains that we tested induced the serine phosphorylation of STAT1 slightly, but none of these strains strongly inhibited IFNγ induced serine phosphorylation of STAT1 (Fig. 3C). These results indicate that ROP16 does not play a role in serine phosphorylation of STAT1 and that type I, II, and III strains do not differentially modulate STAT1 serine phosphorylation. Thus, *Toxoplasma* infection alone can induce low levels of STAT1 serine phosphorylation independently of ROP16 and ROP16 mediates the tyrosine phosphorylation and subsequent nuclear translocation of STAT1, but this nuclear phospho-STAT1^Tyr701/Ser727^ does not seem to be transcriptionally active.

### Type I, II, and III Strains All Inhibit STAT1 Transcriptional Activity

While our results demonstrate that type I, II, and III parasites can all inhibit the IFNγ induced expression of IRF1, we have also shown that type I, II, and III parasites can differentially modulate specific aspects of the IFNγ/STAT1 signaling pathway. The type II GRA15 protein induces IRF1 expression independently of STAT1 (Fig. 1D), and the rhoptry kinase ROP16 induces STAT1 tyrosine phosphorylation (Fig. 2, 3). Additionally, the expression of many IFNγ-regulated genes can be induced by transcription factors other than STAT1; for example the activation of IRF1 by GRA15 via NF-κB (Fig. 1A, B) and the induction of *Socs1* by ROP16 via STAT3 or 6 [25]. It is therefore difficult to interpret the modulation of the STAT1 transcriptional response by different *Toxoplasma* strains using the expression of particular genes as a read out. We instead decided to use a stable STAT1 reporter cell line to determine the ability of the *Toxoplasma* clonal lineages to interfere with STAT1’s activity in the nucleus. One previous report used two different luciferase reporters to demonstrate that infection with *Toxoplasma* inhibits STAT1 transcriptional activity [9]. However, one of these reporters was a stable reporter but comprised the entire CIITA pIV promoter, containing binding sites for IRF1, AP-1, and NF-GMa transcription factors and an E-box site as well as a GAS site, making it difficult to determine whether STAT1 activity itself was being affected by *Toxoplasma* infection or if one of the other transcription factors was being affected. The second reporter measured STAT1 activity more clearly as it contained only a minimal GAS site, however the reporter vector was transiently transfected into cells. Given recent results that indicate that chromatin remodeling factors such as Brahma-related gene (BRG)-1 are differentially recruited to GAS sites after *Toxoplasma* infection to inhibit STAT1-mediated transcription [14], and that *Toxoplasma* infection can modulate chromatin modifications resulting in changes in gene expression [36], a transient plasmid reporter that is not integrated into the genome and does not have a normal chromatin structure also may not be an accurate measure of STAT1 transcriptional activity [37], [38]. Additionally, potential strain differences in the inhibition of these reporters were not investigated.

**Figure 4:**
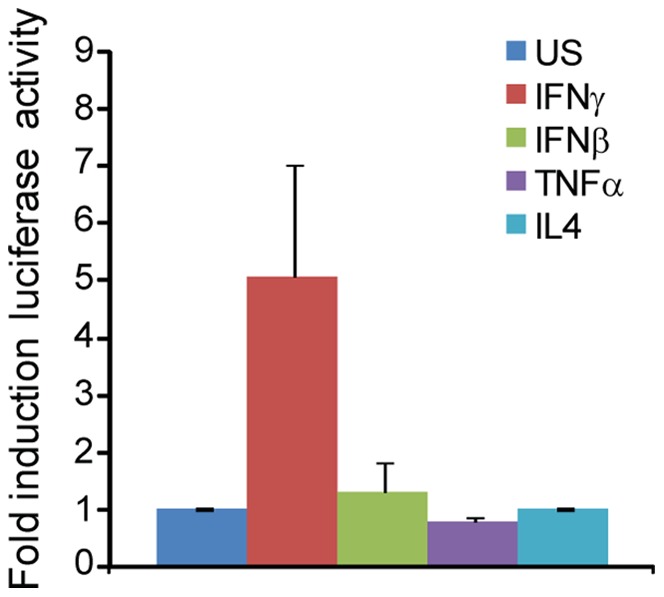
Characterization of HEK293 GAS reporter cell line. A HEK293 GAS luciferase reporter cell line was left unstimulated or stimulated with 100 U/ml IFNγ, 100 U/ml IFNβ, 20 ng/ml TNFα, or 50 ng/ml IL4. Cells were lysed 6–20 hours later and luciferase activity was measured. Average luciferase induction normalized to unstimulated cells from three experiments is shown and error bars represent standard error.

**Figure 5:**
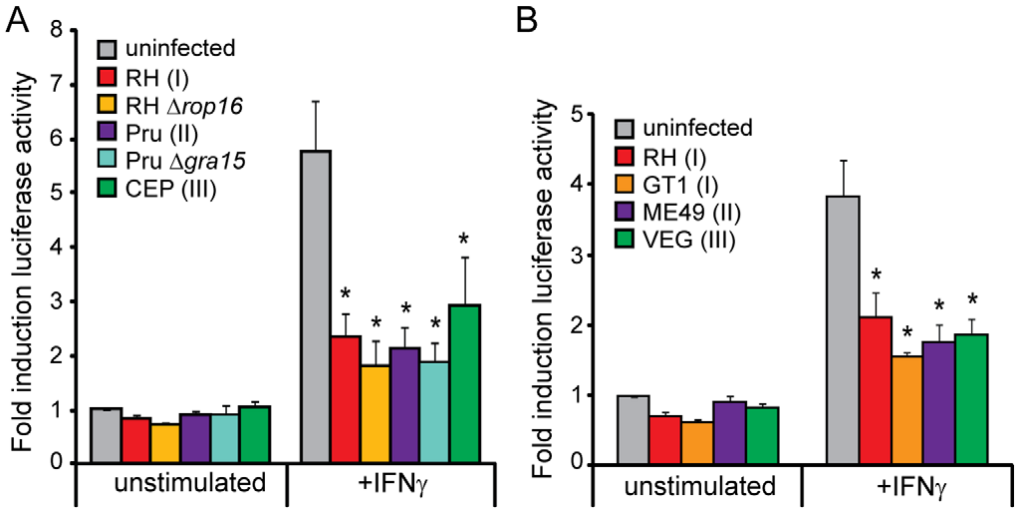
All three clonal lineages of *Toxoplasma* inhibit STAT1-mediated gene expression. A, B. A HEK293 GAS luciferase reporter cell line was infected with RH(I), RHΔ*rop16*, GT1(I), Pru(II), PruΔ*gra15*, ME49(II), CEP(III), or VEG(III) parasites, or left uninfected, and subsequently stimulated, or not, with 100 U/ml IFNγ. Cells were then lysed and luciferase activity was measured. Results are from 2–8 experiments per condition, with a pre-infection time of 1–5 hours followed by a stimulation of 12–24 hours, and represent the average induction over uninfected, unstimulated samples. Error bars represent SEM. Asterisk (*) indicates p<0.05 compared to uninfected cells in the same condition.

We therefore developed a stable GAS reporter cell line in the easily transduced HEK293 cell line to measure STAT1 transcriptional activity directly. Treatment of this GAS reporter cell line with IFNγ, but not IFNβ, TNFα, or IL4, results in the robust induction of luciferase activity (Fig. 4). We infected this cell line with RH(I), RHΔ*rop16*, Pru(II), PruΔ*gra15*, or CEP(III) parasites, stimulated the cells with IFNγ, and measured the induction of luciferase activity. Infection with any of these strains significantly inhibited IFNγ induced luciferase activity, and the extent of this inhibition did not vary significantly between the strains (Fig. 5A). These reporter experiments also confirmed that while ROP16 can activate the tyrosine phosphorylation and nuclear translocation of STAT1 (Fig. 2), this STAT1 is not transcriptionally active; infection of this cell line with any of these strains did not result in the induction of luciferase (Fig. 5A). To verify that this ability to inhibit STAT1 transcriptional activity is common to the type I, II, and III clonal lineages and not just RH(I), Pru(II), and CEP(III) strains, we also infected this cell line with other representative strains from these lineages, GT1(I), ME49(II), or VEG(III), as well as RH(I) (Fig. 5B). Again, all of these strains were able to inhibit IFNγ induced luciferase activity. These results indicate that type I, II, and III strains can all inhibit IFNγ induced STAT1 transcriptional activity to a similar extent.

### Type I, II, and III Strains All Inhibit Global IFNγ Induced Transcription

**Figure 6:**
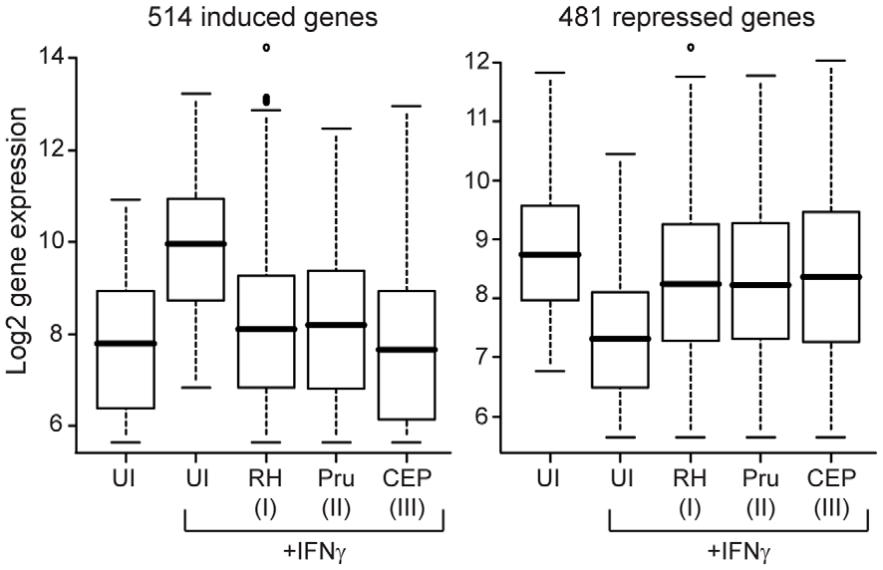
All three clonal lineages of *Toxoplasma* inhibit global IFNγ-induced gene expression. RAW264.7 macrophages were infected with RH(I), Pru(II), or CEP(III) parasites, or left uninfected (UI) for 24 hours with 100 U/ml IFNγ added for the last 6 hours of infection, and host gene expression was analyzed by microarray analysis. Greater than 2-fold IFNγ induced (left) and repressed (right) genes were determined from the uninfected samples. Boxplots are shown of the log_2_ expression of these genes in all samples. Data are from two arrays for the uninfected conditions and one array for each infected sample.

Although all *Toxoplasma* strains that we have tested inhibit a stable GAS reporter cell line, we have seen that *Toxoplasma* strains can differentially affect particular aspects of the IFNγ signaling pathway through GRA15 and ROP16, and it is therefore unclear whether the ability to inhibit STAT1 activity corresponds to the ability of type I, II, and III strains to similarly inhibit global IFNγ induced gene expression. We therefore analyzed the effect of infection with an RH(I), Pru(II), or CEP(III) strain on IFNγ induced transcription using microarray analysis. As more genes have been found to be induced by IFNγ in macrophages than fibroblasts [13], [14], we pre-infected a murine macrophage cell line (RAW264.7) with the above strains for 24 hours, adding IFNγ for the last six hours. We isolated RNA from these cells as well as uninfected control cells, with and without IFNγ stimulation, and analyzed gene expression with Affymetrix microarrays. In this macrophage cell line, 514 genes were more than 2-fold upregulated by IFNγ treatment, while the expression of 481 genes was more than 2-fold repressed (Fig. 6). In the pre-infected samples, 431 of the 514 induced genes were at least 2-fold inhibited by at least one strain, with 314 genes being inhibited by all strains (Fig. 6). Interestingly, the expression of genes that are important for control of *Toxoplasma* infection, *Nos2* [39], *Iigp1/Irga6* [40], [41], *Iigp2*/*Irgm2* [42], and *Tgtp*/*Irgb6* [42] were at least 2-fold inhibited by all three strains. Of the 481 IFNγ repressed genes, the repression of 312 of them was more than 2-fold inhibited by at least one strain while 147 genes were inhibited by all three strains (Fig. 6). It seems then that while *Toxoplasma* strains may differentially inhibit small subsets of IFNγ responsive genes, all three of the clonal lineages significantly inhibit global IFNγ induced gene expression.

## Discussion

The expression level of many genes is regulated by multiple transcription factors allowing more precise control and responsiveness to varying stimuli. While we find that strains representing three *Toxoplasma* clonal lineages, types I, II, and III, can all inhibit IFNγ induced STAT1 transcriptional activity, these strains also differentially modulate certain IFNγ responsive genes through the activity of at least two known polymorphic effectors, GRA15 and ROP16. In studying the ability of *Toxoplasma* to inhibit the IFNγ response, the choice of readout for IFNγ induced gene expression is therefore very important, as some IFNγ responsive genes are also activated by *Toxoplasma* through GRA15, ROP16, and likely other secreted proteins.

GRA15_II_-mediated activation of NF-κB can induce expression of IRF1, and the levels of IRF1 in Pru(II) infected cells stimulated with IFNγ are virtually identical to those of Pru(II) infected cells that were not stimulated (Fig. 1A). This indicates that Pru(II) parasites can inhibit IFNγ induced expression of IRF1, even though they induce IRF1 through GRA15-mediated activation of NF-κB (Fig. 1A, D). Similarly, ROP16_I/III_ induces *Socs1* expression by 10-fold in murine BMDM [25], likely through STAT3 or STAT6. But, our microarray data from the murine macrophage RAW264.7 cell line shows that pre-infection with RH(I) parasites can still inhibit IFNγ induced *Socs1* transcript by two-fold. Thus, although *Toxoplasma* is able to inhibit the STAT1-mediated induction of genes such as *IRF1* and *Socs1*, it does not inhibit the expression of these genes activated by other transcription factors. This indicates that whatever mechanism *Toxoplasma* employs to inhibit the IFNγ-induced transcriptional response must specifically target STAT1-mediated transcriptional activation of genes.

While neither GRA15 nor ROP16 affects the ability of *Toxoplasma* strains to inhibit the STAT1-mediated global induction of IFNγ responsive gene expression, it is unclear how large of an effect GRA15 and ROP16 have on subsets of IFNγ responsive genes as our experiments were done in a different cell line than previous transcriptional analyses on GRA15 and ROP16. However, IRF1 is an important secondary transcription factor in the response to IFNγ. Additionally, NF-κB is likely to co-regulate other IFNγ responsive genes besides IRF1. A significant number of genes induced by both IFNγ and Pru(II) infection are GRA15-regulated (Data S1). While one microarray analysis in HFFs found that IFNγ responsive genes that were also induced by Pru(II) infection alone were associated with TNFα signaling and included many NF-κB target genes [13], another microarray analysis in murine BMDM did not find an enrichment in NF-κB target genes among genes induced by both IFNγ and another type II strain, NTE [14]. However, it is unknown whether the NTE(II) strain has an active copy of GRA15 and activates NF-κB.

The strongest effect of ROP16 on IFNγ signaling seems be on the phosphorylation status of STAT1 (Fig. 2). Since ROP16 directly tyrosine phosphorylates STAT3 and STAT6 [20], [21], it is likely that tyrosine 701 on STAT1 is also a direct target. It seems that either the affinity or catalytic efficiency of ROP16 for STAT1 is lower than for at least STAT6 because clear phospho-STAT1^Tyr^ activation was only observed in cells infected with at least three parasites, whereas only one parasite needs to inject its rhoptry contents into a host cell to activate STAT6 [43].

It is still unclear why we observe a higher level of IFNγ induced phospho-STAT1^Tyr^ after pre-infection with any strain of *Toxoplasma* (Fig. 2A). This phenotype is not dependent on ROP16 as it also occurs in RHΔ*rop16* infected cells. The transcripts of the main components of this pathway, IFNγ receptor 1 and 2, JAK1 and 2, and STAT1, are not upregulated by type I *Toxoplasma* infection in HFFs, according to previous microarray data [19]. Additionally, SOCS proteins that can downregulate JAK and STAT1 phosphorylation are actually induced by *Toxoplasma* infection [12], and the expression of the protein tyrosine phosphatases (PTPs) that are known to dephosphorylate JAK1, JAK2, or STAT1 [44] are not downregulated by infection alone [19].

Our data suggest that the type I, II, and III strains use a similar mechanism to inhibit STAT1 transcriptional activity in the nucleus. This inhibition is independent of how STAT1 is activated; *Toxoplasma* can also inhibit the activity of ROP16 induced phospho-STAT1^Tyr^, and this interference is specific for STAT1, as ROP16-activated STAT3 and STAT6 are transcriptionally active [18], [25]. A recent study, which focused mainly on the IFNγ induced expression of CIITA and MHC class II genes, concluded that *Toxoplasma* inhibits IFNγ induced gene expression through impaired BRG-1 chromatin remodeling [14]. Although that may be the mechanism for CIITA, the IFNγ induced expression of IRF1 does not require BRG-1 remodeling [45]. It is therefore important for future studies to determine the mechanism by which *Toxoplasma* inhibits the STAT1-mediated induction of primary response genes such as *IRF1*.

## Supporting Information

Data S1 **GRA15-regulated IFNγ responsive genes.** 374 genes induced by IFNγ treatment and by Pru infection in a published microarray analysis and also present in a microarray analysis of HFFs infected with GRA15-deficient and GRA15-overexpressing *Toxoplasma* strains are listed. Whether these genes were also found to be at least two-fold GRA15-regulated in at least one parasite genetic background is also indicated.
(XLSX)
